# Temperature and Heat Transfer Control During Freeze Drying. Effect of Vial Holders and Influence of Pressure

**DOI:** 10.1007/s11095-022-03353-4

**Published:** 2022-08-04

**Authors:** Shuai Bai Palmkron, Linnea Gustavsson, Marie Wahlgren, Björn Bergensthål, Anna Millqvist Fureby

**Affiliations:** 1 Department of Food Technology, Engineering and Nutrition, Lunds Universitet, Institutionen För Livsmedelsteknik, Box 124, 221 00 Lund, Sweden; 2 Chemical Process and Pharmaceutical Development, RISE, 114 28 Stockholm, Sweden

**Keywords:** freeze-frying, heat transfer, radiation, sublimation rate, vial holder

## Abstract

**Objective:**

A common issue of freeze drying is the inhomogeneity between samples, both in regards to water content and structure. The purpose of this study is to address this issue, and try to understand the cause of inhomogeneity in the heat transfer and sample temperature.

**Methods:**

The temperature and the heat transfer was measured using different setups, both with and without vial holders at various positions at different shelf temperature and chamber pressures. By comparing sublimation rate measurements (water sample) with temperature equilibrium measurements with a non-evaporating liquid (oil sample), the heat transfer contribution from radiation and conduction could be separated and investigated individually.

**Results:**

The oil sample temperature increases each time the pressure is decreased; the increase is highest at lower shelf temperatures. Using vial holder reduces the deviation between the samples but have limited effect on the temperature increase. The sublimation rate for water sample is pressure dependent and samples close to the walls have a higher sublimation rate than vials in the center. The sublimation rate increases slightly when using a vial holder but the deviation between vials becomes more random.

**Conclusions:**

The heat transfer consists of conduction through rectified vapor and radiation from surrounding walls, about 65–75% of the heat is transferred by conduction and 25–35% by radiation under normal operational conditions. As the vial holder is also influenced by the radiation, the vial inside the holder is indirectly affected by the surrounding radiation.

**Supplementary Information:**

The online version contains supplementary material available at 10.1007/s11095-022-03353-4.

## Introduction

In the pharmaceutical and biotechnology industries, freeze drying is commonly used to obtain products that have long-term stability. A successful freeze-drying process enablesgentle drying that results in a product with low water content, maintained structure and good rehydration properties. A key problem encountered in freeze-drying is the limited temperature and heat transfer control during the process caused by uneven heat transfer inside the freeze dryer, resulting in inhomogeneity between vials standing on different positions on the shelf during the drying process. This is an issue especially noticeable when freeze drying at low chamber pressures[1, 2]. The knowledge of how pressure influences heat transfer and sample temperature can be essential when designing a functional freeze-drying program.

Freeze-drying is conducted in three or four steps; freezing,(annealing), primary drying and secondary drying The quality of the freeze dried product is affected by the temperature in all steps but especially the temperature during the primary drying in correlation to material properties of the formulation.

The collapse temperature, ${\mathrm{T}}_{\mathrm{c}}$, is the maximum allowable temperature for an amorphous formulation to keep its macroscopic structure during freeze-drying to generate a porous cake. [3, 4] ${\mathrm{T}}_{\mathrm{c}}$ is thus crucial when designing a freeze-drying program. Drying formulations with low ${\mathrm{T}}_{\mathrm{c}}$ can be challenging as this requires a low sample temperature, usually obtained by a low shelf temperature and a low chamber pressure. The influence of low pressure and low shelf temperatures on the heat transfer is unfortunately often overlooked, resulting in uneven heat transfer and loss of temperature control that can lead to inhomogeneous drying and loss of reproducibility. There are previous studies focusing on heat transfer during freeze-drying and how vial holders and radiation shields can help solve issues such as inhomogeneous drying [1, 5–7].

The heat transfer to the sample can occur in three different ways, direct contact conduction between the shelf and the vial, conduction or convection through the rarefied vapor, and by radiation. Direct contact conduction between shelf and vial transfers heat occurs by the molecular vibrations in the solid matter. As the surfaces of the vials and the shelves are not perfectly flat, the contact points of the solid vial and the shelves are almost negligibly small. The heat transfer *via* direct contact conduction is not affected by pressure and thus constant during freeze drying. Conduction or convection through the rarefied vapor over the thin gap between shelves and vial depends on the conditions determined by the distance of the gap, the temperature difference, and the pressure [5].Convective transfer occurs if the Rayleigh number (Ra, the ratio between the timescale of heat transport through diffusion and convection) is significant, Ra > 1800 [7]. Under typical conditions of the freeze-drying of vials, the characteristic distance is less than a millimeter, and Ra becomes around 1. Thus, the heat transport is clearly conductive under these conditions.

The conductive heat transport through a thin vapor layer can be described in relation to the Knudsen number (Kn, the ratio between the free path of a molecule and the characteristic dimension of the heat transfer). At Kn < 0.01, the gas may be treated as a continuum and can be described by the heat conductivity of the gas. The heat transfer is weakly dependent on temperature, independent of pressure, but dependent on the distance within the continuum regime. At Kn above 10, direct molecular collisions describe the conductivity. In this molecular regime, the conductivity is linearly dependent on the pressure but independent on the distance. At intermediate Kn (0.01 < Kn < 10), the conductivity is considered to be in a transition regime [5, 8, 9].

Radiation is the heat transfer by the emission and absorption balance of electromagnetic radiation. The thermal radiation comes from all available surfaces, thus not only from the temperature-controlled shelves. As the pressure inside the chamber is reduced, the heat transfer through radiation becomes more critical, and the radiation from the surrounding surfaces becomes more influential [1]. The vials on the perimeter of the shelf are more affected by the radiation from the door and walls than vials in the middle of the shelf, resulting in deviations in heat transfer between different positions.

The heat transfer from the freeze-dryer’s chamber to the sample can be described as an apparent heat transfer coefficient, ${\mathrm{K}}_{v,app}$. The coefficient is a sum of the three different modes of heat transfer and defined as the ratio of heat flow to the temperature difference between a heat source and heat sink [10]. ${\mathrm{K}}_{v,app}$ can be experimentally obtained by drying aqueous samples without solids by assuming a semi-constant temperature gradient in the sample and that all transferred heat can be considered consumed by sublimation [1, 2, 6–8, 13–15].

1 $${K}_{v, app}={~}^{{H}_{ sub}}\!\left/ \!{~}_{({T}_{shelf}-{T}_{b})}\right.$$

where, ${\mathrm{T}}_{shelf}$, is the shelf temperature, ${\mathrm{T}}_{b}$ is the temperature at the bottom of the sample, and ${\mathrm{H}}_{sub}$ is the heat flux consumed by the sublimation.

The ${\mathrm{K}}_{v,app}$ has been calculated in several other studies. These authors have studied the heat transfer and sample temperature of sublimating formulations. These studies have focused on the total heat transfer, and have not separated the contribution of radiation from the conduction through the rarefied vapor, and therefor have not been able to determine the source to the inhomogeneous heat transfer. [1, 5, 11–15].

In this study we have compared sublimating with non-sublimating conditions with the objective to determine the radiative and conductive heat transfer contribution to vials in freeze drying. We also investigate how different set-ups influence the different heat transfer contributions and the vial-to-vial homogeneity in freeze drying. The results allow for a separation of the conductive heat transfer from radiative heat transfer. Thus, it has become possible to specifically follow the pressures influence on conductive heat transfer during freeze drying. The present study also aims to investigate the possibilities to improve the temperature control by using vial holder.

## Material and Method

### Freeze Dryer Setup

The experiment is conducted using an Epsilon 2-6D LSCplus freeze dryer (Martin Christ, Germany), operating under controlled pressure and shelf temperature. The lowest possible shelf temperature is -50 ± 1°C. The ice condenser is located in a second chamber, and the condenser temperature is kept constant at -88°C. The freeze dryer has four temperature sensors and three different pressure gauges Piezo, Capacitance, and Pirani. The vials used are 8 ml tubular glass vials with rubber stoppers (VCDIN8R SCHOTT, Germany), the outer diameter of the vial is 22 ± 0.2 mm.

### Freeze Drying Program

For the oil sample temperature equilibrium experiment, the shelf is first cooled from 20°C to -45°C at a rate of 1°C/min and maintained at -45°C for 30 min. The shelf temperature is then raised to either -40°C or -10°C depending on the experiment, the wide temperature range was chosen to clarify how shelf temperature affects the sample equilibrium temperature and heat transfer balance even though -40°C is rarely employed during primary drying. The pressure is then decreases to 200 Pa, and a sequence of pressure changes is started. The pressure is reduced by 50% every 2 h in 6 steps until the vacuum reaches 3.12 Pa (200, 100, 50, 25, 12.5, 6.25, and 3.12 Pa). The same freezing steps were used for the mass flux experiment. The heat transfer was determined batch-wise at the pressures 50, 25, 12.5, 6.25, and 3.12 Pa at -15°C shelf temperature. Pressures below 200 Pa were controlled using the capacitance vacuum gauge, and at pressures above 200 Pa, a Pirani vacuum gauge was used. The shift in pressure gauge caused some pressure fluctuations between 200 and 100 Pa.

### Sample Temperature and Heat Transfer Experiments

**Fig. 1:**
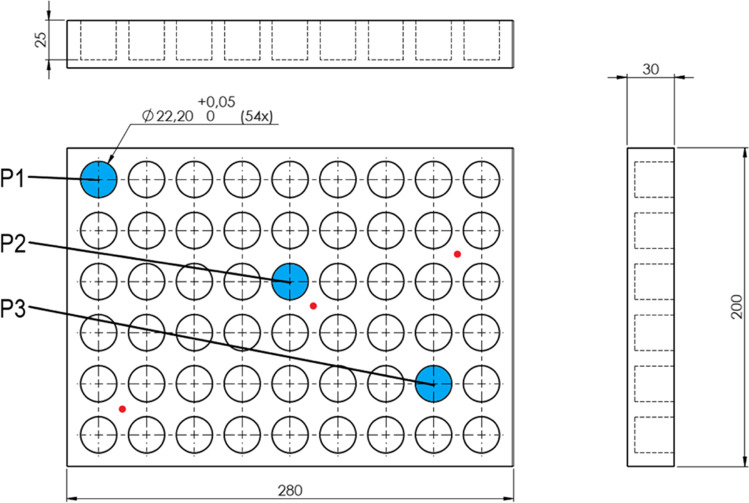
Design of custom-made aluminum vial holder used in setups 2 and 3. The sample temperature is measured with a temperature probe inside the vials placed at positions marked blue. P1 is close to the door of the freeze dryer, and P3 is close to the condenser. The temperature probe can also be inserted into the vial holder (marked with red) to measure the temperature of the vial holder.

The sample temperature was investigated by measuring the temperature of a non-sublimating sample using medium chain triglyceride oil, hereafter termed MCT oil (Miglyol 812, Caesar & Loretz, Germany) or a sublimating sample of pure water. Samples of oil were filled to 2 ml which is the typical fill volume for the vials used (around 1 cm depth),due to sublimation the water sample was filled to 4 ml to ensure that the temperature probes were in contact with the ice during the entire drying step. The sample temperature of three vials and the vial holder temperature were measured using the freeze dryer's temperature probes according to Fig. 1. The temperature was monitored for 2 h at each pressure. The average sample temperature of each setup is presented with the standard deviation given as error bars.

For the heat transfer experiment, the sublimation flux, $\dot{m}={~}^{\Delta m}\!\left/ \!{~}_{\Delta t}\right.$, was determined gravimetrically, assuming that the mass and heat transfer becomes stationary reasonably fast. The results are averages of 54 vials filled with 4 ml of water. The freeze-drying program was interrupted after each pressure step. The samples were weighted, water was refilled and, the program restarted by refreezing the sample at a rate of 1°C/min to -45°C, and the drying was resumed at the next pressure level.

The investigation of sample temperature and heat transfer was conducted using three different setups, as presented in Table I.

**Table I:** The Heat Transfer and Sample Temperature of the Sample Were Investigated Using these 3 Different Experimental Setups

Setup	Description
1. Only Vials	Vials are standing directly on the cooling shelf
2. With vial holder	Vials were placed inside an aluminum vial holder set on the cooling shelf
3. Vial holder with oil	MCT oil was added on the cooling shelf and inside the holes of the vial holder before inserting the vials

The vial holder used in setups was custom made in blank aluminum with 25 mm deep holes and 0,05 mm clearance between vial and holder. The holes are spaced at 8 mm between vials. The vial holder covers the entire freeze dryer shelf. See Fig. 1.

## Results

### Sample Temperature of the Non-Sublimating Sample

**Fig. 2:**
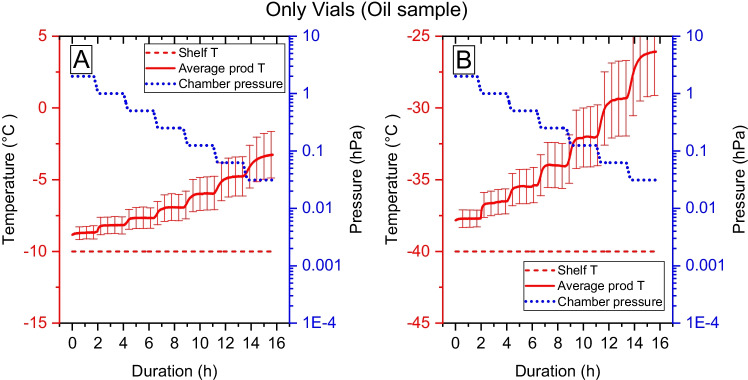
The average sample temperature and the standard deviation between samples of oil using the conventional setup when vials are standing directly on the shelf. Figure A shows the sample temperature increase when pressure is lowered for samples standing on the -10°C shelf, and figure B show the sample temperature at the -40°C shelf temperature.

**Fig. 3:**
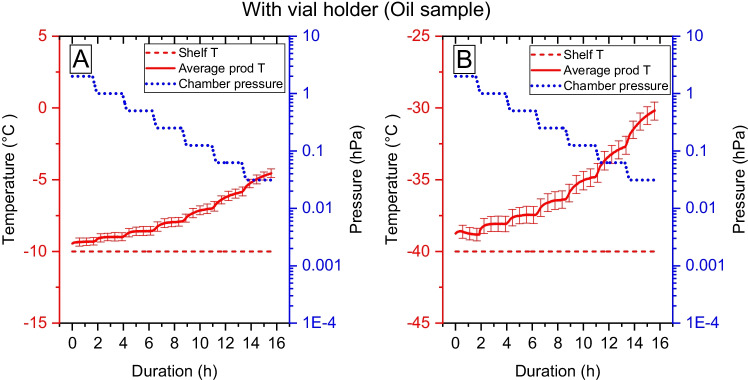
The average sample temperature and its standard deviation of oil when vials are placed inside a vial holder. Figure A shows the sample temperature increase when pressure is lowered for samples standing on the -10°C shelf, and figure B show the sample temperature at the -40°C shelf temperature.

**Fig. 4:**
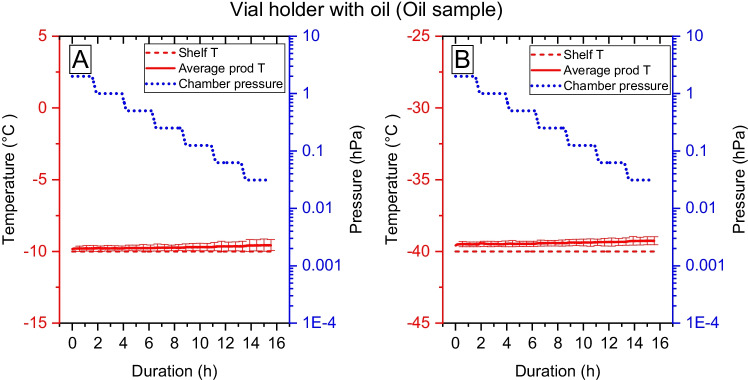
The average sample temperature and its deviation of oil when vials are placed inside a vial holder with oil applied at the cooling shelf and between the vial and vial holder. Figure A shows the sample temperature increase when pressure is lowered for samples standing on the -10°C shelf, and figure B shows the sample temperature increase at the -40°C shelf temperature.

The temperature profiles for the experiments using MCT oil as samples are presented in Figs. 2, 3, 4. The experiment shows the temperature of the sample at different pressures under conditions when no mass and heat are removed by sublimation.

The change in the average temperature for samples containing oil using the setup vials standing on the shelf is presented in Fig. 2. The average sample temperature reaches an equilibrium value after a change in pressure. The temperature of the samples is always higher than the shelf temperature, and the difference between sample temperature and shelf temperature increases each time the pressure is decreased. At the pressure of 3.12 Pa, the average temperature difference between shelf and sample is 7°C when the shelf temperature is -10°C, and 14°C with a shelf temperature of -40°C. The difference is more pronounced for samples standing close to the door than for samples in the middle of the shelf and close to the condenser. The error bars show the between sample variation (n = 3). The individual sample temperatures are given in supplementary material Figure S1.

As described in Table I, two different methods to increase the contact between freeze drying shelf and vial using a vial holder were used. The results can be seen in Figs. 3 and 4. When the vials are inserted into a vial-holder, the temperature increase is reduced, and the deviations in sample temperature between sample positions are reduced, compared with the setup with vials on the shelf, see Fig. 3. At the pressure of 3.12 Pa the average temperature difference between shelf and sample is 5°C with a shelf temperature of -10°C, and 10°C with a shelf temperature of -40°C. The impact of vial position of individual vials and the effect of vial holders are shown in supplementary material Figure S1. However, the difference in temperature between samples and shelves is still large at low pressures.

The third set up, Fig. 4, is a conformation where the heat transfer between shelf and vial-holder as well as between vial and vial-holder is improved by filling the space with MCT oil. The temperature inside the aluminum vial-holder was unaffected by the decreasing pressure see supplementary material S2. The temperature of an oil sample standing on a -40°C shelf at 3.12 Pa pressure has a temperature increase of only 0.7°C. Also, the deviation between different vials in different positions becomes small. It was challenging to manage the experiment with this setup in a reproducible way, since air or vapor bubbles were a constant issue as even small air pockets will expand at low pressures resulting in loss of contact. The varying oil coverage between vials caused the largest deviations in the sublimation rate. Thus, reproducibility was lost, and no further evaluation could be performed. Thus, no results from this setup are further reported in this study.

The summary of the average temperature increases of the different setups is presented in supplementary material Figure S3. The magnitude of the temperature increase is higher at low pressures, and each measure to enhance contact between the vial and the shelf improves the temperature control.

### Sublimation of Water as a Function of Pressure

**Fig. 5:**
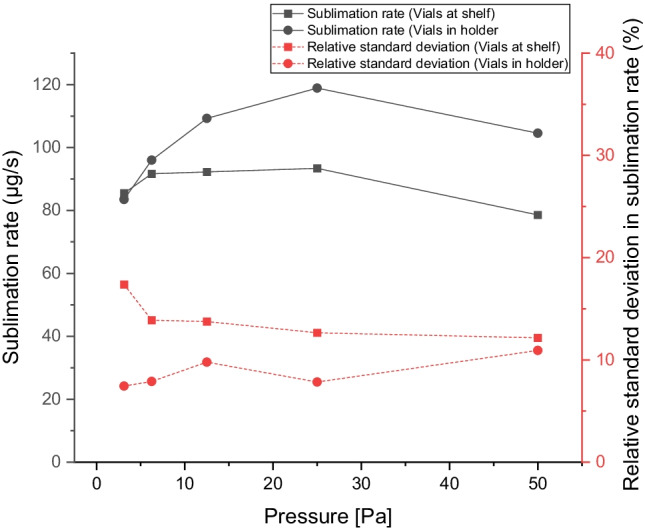
Sublimation rate [µg/s ·vial] as a function of chamber pressure [Pa] and the relative standard deviation [%]. Results obtained with vials at the shelf are marked with squares, and vials in a vial-holder are marked with dots. The shelf temperature is -15°C. The result is an average of 54 vials measured over 2 h of drying time.

Sublimation trials with free vials on the shelf and vials in vial-holder were performed. The results and the relative standard deviation are presented in Fig. 5. It can be noted that the sublimation rate depends on the pressure, reaching a maximum at 25 Pa. A more pronounced pressure dependence is observed when using a vial holder rather than placing the vials directly on the shelf.

**Fig. 6:**
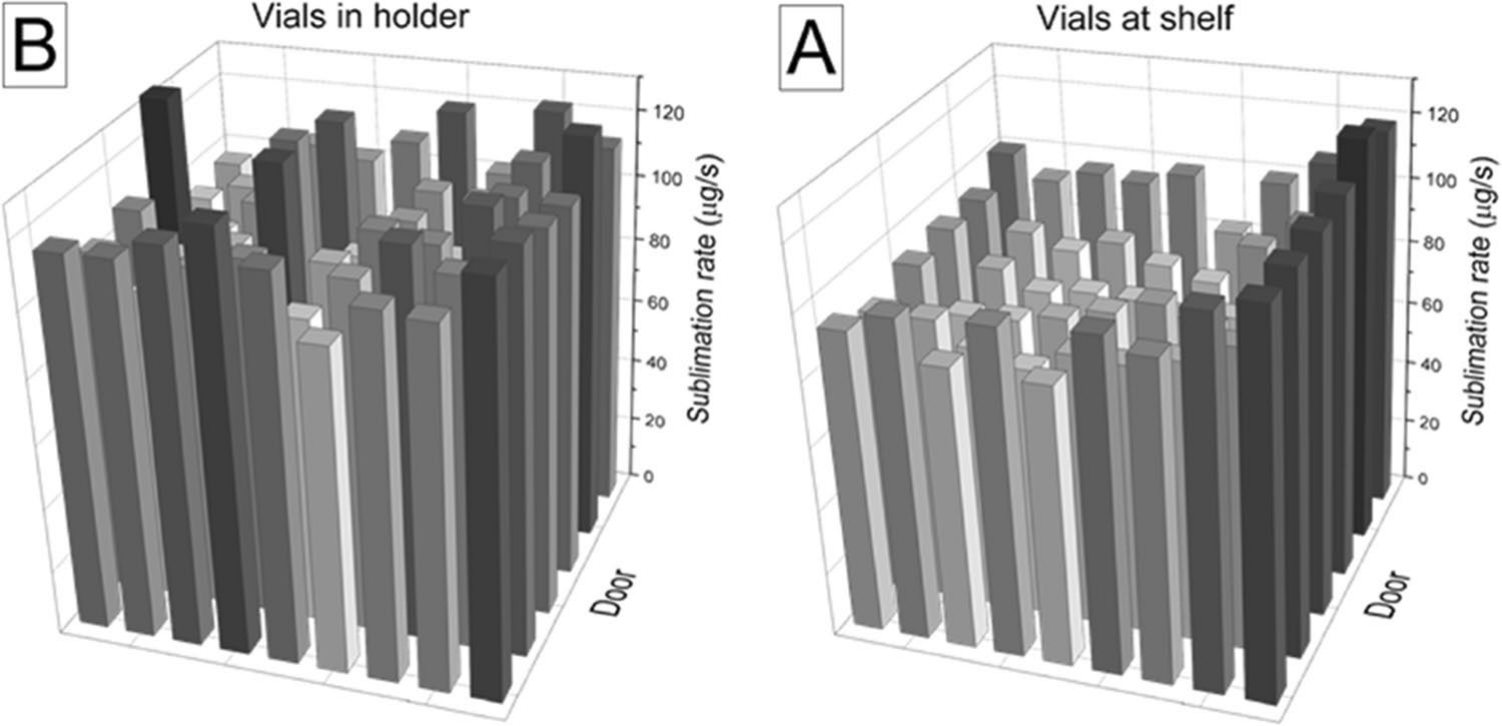
The sublimation rate of individual vials standing directly on the shelf, **A**, and vials standing in a vial-holder, **B**. The shelf temperature is -15°, **C** and the pressure is 12.5 Pa.

The variation in sublimation rate at the pressure of 12.5 Pa is shown in Fig. 6 and depends on the position on the shelf or in the vial holder. The experiment with the samples on the shelf displays a variation depending on the position, between 76 and 119 with an average of 92 µg/s. Samples close to the walls and in particular close to the door have a higher sublimation rate than the vials in the center. In setup 2 the variation (between 94 and 134 with an average of 109 µg/s) is more random. No noticeable wall effects can be seen. Vials on the shelf have higher variability than vials in the vial-holder. It can be observed that the variability between samples on the shelf is somewhat reduced at increasing drying pressure, while for samples in the vial-holder, it is slightly increasing with an increasing drying pressure.

## Discussion

### Heat transfer During Freeze-Drying Separated in Conduction and Radiation

The sublimation consumes heat. The heat is transferred from the shelves by conduction and by radiation from the surrounding walls and door. Thus, for the experiment with water, the heat balance can be written as:

2 $${\dot{\mathrm{H}}}_{\mathrm{ sub},\mathrm{w}}={\dot{\mathrm{H}}}_{\mathrm{ cond},\mathrm{w}}+{\dot{\mathrm{H}}}_{\mathrm{ rad},\mathrm{w}}$$

In our experiment, the sublimation rate was measured gravimetrically. The sublimation heat flux is obtained by ${\dot{\mathrm{H}}}_{\mathrm{ sub},\mathrm{w}}={\dot{\mathrm{m}}}_{\mathrm{ sub},\mathrm{w}}\cdot \Delta {\mathrm{H}}_{\mathrm{ sub}}$ where ${\dot{\mathrm{m}}}_{\mathrm{ sub},\mathrm{w}}$ is the sublimation flow, and ${\Delta \mathrm{H}}_{\mathrm{sub}}$ is the sublimation energy. ${\Delta \mathrm{H}}_{\mathrm{sub}}$ was determined to be 2840 kJ/kg from the vapor pressure curve using the Clausius-Clapeyrons equation for each condition. The data $\mathrm{T}$,${\dot{\mathrm{m}}}_{\mathrm{ sub},\mathrm{w}}$ and ${\dot{H}}_{\mathrm{ sub},\mathrm{w}}$ are provided in the Table S5 in supplementary material. All the calculations of T are done in Kelvin and presented in Celsius.

In the absence of sublimation, the oil experiments are quite different from experiments with sublimating water. The temperature of the samples becomes higher than the shelf temperature, particularly when the pressure is low. The magnitude of temperature differences is prominent, up to 15°C at a shelf temperature of -40°C at 3 Pa. Thus, the heat transfer through contact between the shelf and the vials cools the sample. At the same time, heat is absorbed by radiation from other surfaces in the chamber. A stable temperature is obtained when the heat flux through radiation from the surrounding surfaces becomes equal to the heat transfer by conduction.

3 $${\dot{\mathrm{H}}}_{\mathrm{ cond},\mathrm{oil}}={\dot{\mathrm{H}}}_{\mathrm{ rad},\mathrm{oil}}$$

The heat transferred by conduction is assumed to be described by an energy transferring constant,

${\mathrm{K}}_{\mathrm{v},\mathrm{cond}}$ and the temperature difference between shelf and the bottom of the material in the vial, ${\Delta \mathrm{T}}_{\mathrm{b},\mathrm{ref}}$. Ref refers to type of sample for the experiment (e. g. water or oil), which implies that K_v_ is independent of the content of the vial.

4 $${\dot{\mathrm{H}}}_{\mathrm{ cond},\mathrm{ref}}=\Delta {\mathrm{T}}_{\mathrm{b},\mathrm{ref }}\cdot {\mathrm{K}}_{\mathrm{ v},\mathrm{cond}}$$

The temperature during sublimation of water is lowest at the sublimation front,${\mathrm{T}}_{\mathrm{sf}}$, and highest at the shelf,${T}_{shelf}$. We may divide the temperature drop into two parts, $\Delta {T}_{ice}$, describing the temperature drop over the ice, and the temperature drop over the bottom of the vial over shelf, ${\Delta \mathrm{T}}_{\mathrm{b},\mathrm{w}}$:

5 $${\mathrm{T}}_{\mathrm{shelf}}-{\mathrm{T}}_{\mathrm{sf}}=\Delta {\mathrm{T}}_{\mathrm{ice}}+\Delta {\mathrm{T}}_{\mathrm{b},\mathrm{w}}$$

The temperature drop over the ice layer is estimated assuming that the heat conductivity can be described by a single constant and that the thickness is constant over the experiment.

6 $${\Delta T}_{\mathrm{ ice}}={~}^{{\dot{H}}_{sub,w}}\!\left/ \!{~}_{({\lambda }_{ice}\bullet \frac{A}{L})}\right.$$

where ${\uplambda }_{\mathrm{ice}}$ is the heat conductivity of ice, A is the cross-section area of the vial, and L is the thickness of the ice. However, the expression is an approximation, partly because the ice layer may be inhomogeneous, L changes over the experiment, and eventual heat conduction through the vial wall is neglected.

In the oil experiment, the temperature difference is obtained as:

7 $${\mathrm{T}}_{\mathrm{ oil}}-{\mathrm{T}}_{\mathrm{ shelf}}={\Delta \mathrm{T}}_{\mathrm{ b},\mathrm{oil}}$$

where ${\mathrm{T}}_{\mathrm{oil}}$ is the equilibrium temperature of the oil, $\Delta {T}_{b,oil}$ is the temperature difference over the bottom of the vial in the oil experiment. It is assumed that the temperature of the liquid oil in the samples are uniform.The heat transfer between the walls and the sample by radiation is assumed to be estimated by a system constant, ${\mathrm{c}}_{\mathrm{rad}}$:

8 $${\dot{\mathrm{H}}}_{\mathrm{ rad},\mathrm{ref}}=\left\{{T}_{wall}^{4}-{T}_{average, ref}^{4}\right\}\bullet {\mathrm{c}}_{ rad}$$

where the driving force for the radiation is an exponential difference between an assumed value for the walls (288 K), ${T}_{wall}$ and a temperature average for the ice ${\mathrm{T}}_{average,ref}={\mathrm{T}}_{\mathrm{sf}}+\frac{\Delta {\mathrm{T}}_{\mathrm{ice}}}{2}$ have been used. For the oil experiments, it is assumed that the sample temperature can be used as a ${\mathrm{T}}_{\mathrm{average}}$. The constant ${\mathrm{c}}_{\mathrm{rad}}$ includes geometrical features, absorption properties of the radiation as well as the universal radiation constant, and it is assumed to be equal in the experiment with water and with oil.

The ${\mathrm{K}}_{\mathrm{v},\mathrm{cond}}$ can be assumed to be equal to both oil and water experiment as this is the conductivity of surrounding materials. As for ${\mathrm{c}}_{\mathrm{rad}}$ the emissivity of water and oil are different, but as the main radiation exchange is between the vial and the surroundings, the heat is then transferred from the vial to the sample *via* conduction. Thus we may assume that ${\mathrm{c}}_{\mathrm{rad},\mathrm{oil}}$=${\mathrm{c}}_{\mathrm{rad},\mathrm{w}}$.

The system can be solved by combining Eqs. 2 with data from the water experiment and Eq. 3 with data from the oil experiments and using the relations for the temperatures (Eq. 5–7), the conduction (Eq. 4), and the radiation (Eq. 8). The interesting aspect is that the contribution from radiation and conduction can be separated. The derivation of the expressions for conductive heat transfer coefficient ${K}_{v, cond}$, and the radiative heat transfer constant, ${c}_{rad}$. Equation (9) and (10) is presented in the supplementary material S6.

9 $${K}_{v,cond}=\frac{{\dot{H}}_{sub,w}}{\Delta {T}_{w}+\Delta {T}_{oil}\bullet \frac{\left\{{T}_{wall}^{4}-{T}_{average,w}^{4}\right\}}{\left\{{T}_{wall}^{4}-{T}_{average,oil}^{4}\right\}}}$$

10 $${c}_{rad}=\frac{{\dot{H}}_{sub,w}-{K}_{v,cond}\bullet \Delta {T}_{b,w}}{\left\{{T}_{wall}^{4}-{T}_{average,w}^{4}\right\}}$$

**Fig. 7:**
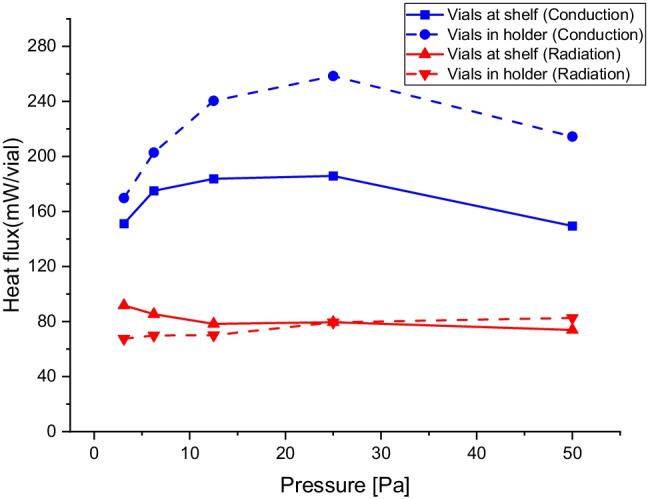
Analyses of the heat flux when sublimating pure water distributed over a contribution from conduction from the shelves and radiation from the surrounding walls. A. Sublimation heat flux in absolute units [mW/vial] separated into heat transfer by conduction ($\dot{{H}_{cond}},$ blue), and heat transfer by radiation from the surrounding walls ($\dot{{H}_{rad}},$_,_ red). The experiment using vials at the shelf is shown with a continuous line, and the experiment with a vial holder is shown with a dashed line. The data is obtained by solving Eqs. 2–10, using the results in Figs. 2 and 3. The values are an average of data from the -10 and -40°C shelf temperatures in Eq. 9.

Using Eqs. 9, 10 and the data from the oil experiments in Fig. 2, 3, and the data from the drying of the water sample in supplementary material Table S5, the fraction of the heat flux originating from the conduction at the shelf and the radiation from the surrounding during the drying of water can be determined. The results are shown in Fig. 7.

The data is calculated based on an average of the values obtained from the oil experiment with -40°C and -10°C. The differences in ${\mathrm{K}}_{\mathrm{v},\mathrm{cond}}$ were reasonably small (using Eq. 9) using data from both temperatures (3% for vials at shelf and 5% for vials in vial-holder).

The result shows that the heat flux from the radiation (${\mathrm{H}}_{\mathrm{rad}}$) has an equal magnitude when the vial-holder is used and without it (Fig. 7). The limited shielding effect is primarily due to that the vial holder also receives radiation form the surrounding walls. As shown in Figure S2 in supplementary material, the vial-holder temperature increases with decreasing pressure in the same magnitude as the oil samples without vial-holder. This suggest that even if the vial holder is shielding the vials from direct radiation from the walls, the vials receives equal amount indirectly from the vial holder. The vial-holder adds to the conductive heat transfer that reaches a maximum at a pressure of 25 Pa. However, there is no dramatic difference using a vial-holder; the obtained increase in the conductive heat transfer is about 30%. The results also show that the heat flux by radiation is important and contributes to about 25% to 35% of the heat consumed by the sublimation process. It can be noted that the range of results obtained for vials on the shelf scatters between values 20% below and 20% above the average, and are highly dependent on the location of the sample, Fig. 6A, which agrees with an interpretation that an important part of the heat transfer is through radiation.

**Fig. 8:**
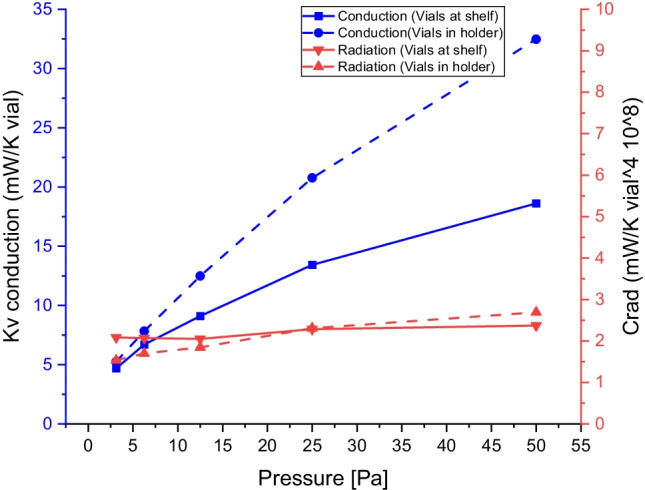
Heat transfer coefficient for the heat conduction (K_v, cond_) and the radiation heat transfer constant (C_rad_) as a function of pressure. Continues curve represents experiments with vials at the shelf and dashed line experiments using a vial-holder. They were determined using Eqs. 9,10, and data from table 2 in supplementary material and Figs. 2 and 3. The values are an average of data from the -10 and -40°C shelf temperatures in Eq. 9.

The conductive transfer coefficient,${\mathrm{K}}_{\mathrm{v},\mathrm{cond}}$, and the radiation constant is shown as a function of the pressure in Fig. 8. It can be seen that that the transfer coefficient, ${\mathrm{K}}_{\mathrm{v},\mathrm{cond}}$, increases on increasing pressure and that it is higher with the vial holder. The curve shape (approaching a linear pressure dependence at very low pressures and showing a decreased gradient at higher pressure, theoretically tending towards a plateau value) agrees with an interpretation that the experiment is performed at a pressure when the heat transfer is in a transition region between conduction through direct molecular collisions (Kn > 10), and when the conduction can be estimated according to the continuum model from a heat transfer coefficient of the vapor (Kn < 0.1).). The Knudsen number in the experiment ranged from about < 1 for vials on the shelf at higher pressure to 10 with the low pressure and vial holder. The conduction through molecular collisions is expected to be linearly dependent on the pressure, while the conduction in the continuum model is independent of the pressure. The molecular collision model and the continuum model are usually combined into a transition model for intermediate Kn [16]. The heat transfer coefficient according to the transition model may be 3 mW/K and vial at low pressure (3 Pa) and around 15 mW/K at high pressure (50 Pa) for a vials on the shelf. The transfer coefficient at low pressure is lower than the experimental observation of 5 mW/K but in agreement at high pressure 18 mW/K (Fig. 8). Similar estimations for a vial holder would give around 5 mW/K at 3 Pa and 29 mW/K in good agreement with the experimental observations (Fig. 8). Also the sublimation rate was modelled and agreed well with the experimental data. For vials on the shelves a maximum sublimation heat flow of 150 mW/vial was observed at 20 Pa followed by a shallow decrease down to 140 mW/K at 50 Pa. For the vials in the vialholder a maximum sublimation heat flow of 220 mW/vial was observed at 18 Pa followed by a shallow decrease down to 170 mW/K at 50 Pa. The transition equation and the results of the modelling of the heat transfer are displayed at S7 and of the sublimation heat flow at S8 in the supplementary material. However, these models demand a few parameters that are difficult to estimate. The free molecular model includes an accommodation parameter [16], and the continuum model is quite sensitive to the distances [5] Here we used 1 for the accommodation parameter and a comparable thin average distance of 0.25 mm. For the vial-holder the theoretical estimations are more complicated as there are two gaps to consider. However, the magnitude and the shape of the results seem to somewhat agree with what is expected from the theory.

The constant for the heat transfer through radiation, Fig. 8, appears slightly increasing with increasing pressure (if compared with the ${\mathrm{K}}_{\mathrm{v}}$). This observation supports that it is possible to estimate the two contributions of the energy transfer by using the simplified approximations in terms of the geometrical and surface temperature effects of the freeze dryer.

### Control of the Temperature of Sublimating and Non-Sublimating Sample

The results show that the control of the sublimation is dependent on heat transfer by conduction (65–75%) and radiation (25–35%). It is expected that the conduction through the rarified vapor is relatively reproducible. However, the heat transfer through radiation to samples in free vials may vary.

**Fig. 9:**
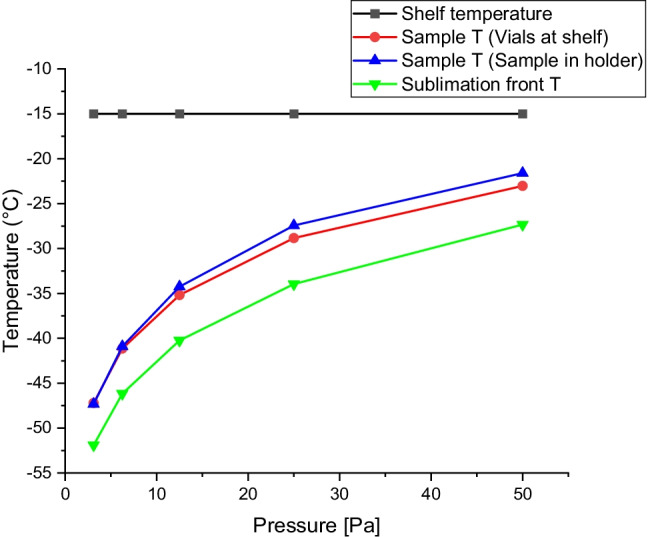
The temperature at the shelf (black line), in the bottom of the sample (red line for vials at the shelf and blue line for vials in vial holder), and at the sublimation front (green line) given as a function of drying pressure. The results are estimated from the K_v, cond,_ and c_rad_ determined in Fig. 8.

The temperature during the primary drying is modeled by Eqs. 5–7. The temperatures are shown in Fig. 9 as a function of pressure for vials at the shelf and vials in vial-holders. It is clear that the drying pressure primarily determines the temperature. Although, if the shelf temperature is raised, the heat flux is expected to increase, and the vial bottom temperature differs more from the sublimation front temperature. The influence of radiation from door and walls affect the drying rate, resulting in deviation at the end of primary drying between various positions resulting in differences in water content in the final product, being a major issue for the industry [17].

In secondary drying, the drying flux is much lower. Thus, the sample temperature is expected to rise as the heat flux for vaporization gradually becomes smaller. As shown in the oil experiments, the temperature might be due to radiation rising above the shelf temperature.

The shelf temperature also influences the sample temperature increase. A lower shelf temperature means a larger difference in temperature between the walls/door and shelf (further from equilibrium) (Figs. 2 and 3). Therefore, a low shelf temperature results in larger relative temperature differences.

There are several purposes of having a vial holder: providing improved contact to the cooling shelf, keeping a homogenous temperature within the vial holder, and shielding the vials from radiation. However, this study shows that a vial holder neither maintains a very homogeneous temperature for non-sublimating samples, Fig. 3 nor a very homogeneous sublimation rate for sublimating samples, Figs. 5 and 6. Although, it can be noted from Fig. 5 that the variability is reduced by about 50% when the vial holders are used compared to without them.

This deviation for vials at the shelf is mainly caused by the difference between vials close to the door or walls and in the center of the shelf (Fig. 6). The variation between samples increases as the pressure decreases (Fig. 5). When pressure is low, the heat transfer from radiation becomes relatively more important and affects the results stronger (Fig. 7) in agreement with an increase in variation between samples.

However, for a sublimating sample in the vial-holder, the deviations in sublimation rate are more random, and no obvious pattern can be observed. The variation is observed to be increasing with increasing pressure (Fig. 8). As the samples are mostly isolated from the surroundings by the vial holder, it is safe to assume that the heat transfer is affected mainly by variations in the contact between vial and vial holder, as there is a strong correlation between snug fitting vials and high sublimation rate. This interpretation agrees with the smaller contribution (in relative terms) of the radiation to the total heat transfer (Fig. 7). It was also noticed that even the smallest amount of moisture between contact surfaces enhanced the sublimation rate significantly, most likely as this would increase the heat transfer by conduction. At low pressures, the molecular transfer mechanism dominates, making the distance dependence smaller and thereby the fitting issue smaller, explaining the reduced variation at very low pressures.

## Conclusions 

The study shows that temperature control and heat transfer control are challenging tasks in freeze-drying. Temperature control is vital for preventing sample collapse during freeze-drying. Heat transfer management is crucial to ensure homogenous drying conditions where all samples end up with the same water content at the end of secondary drying.

The heat transfer consists of both a conduction contribution through a thin layer of rarified vapor as well as a radiation contribution from surrounding walls.

By comparing sublimation rate measurements with temperature equilibrium measurements for a non-evaporating liquid, it was possible to separate the heat transfer through conduction from the heat transfer from radiation. The total heat transfer was higher when using the vial holder, and the increase was due to improved conduction. The results showed that about 65–75% of the heat is transferred by conduction and 25–35% by radiation under normal operational conditions. The ratio showed a limited dependence on the investigated pressure range (3–50 Pa). This shows how big influence radiation have on freeze drying, and can be especially relevant when freeze drying samples with bad contact to the shelf such as with syringes [12] and pellets. The knowledge gained from this study can influence new ways to solve issues with radiation or utilizing radiation in freeze drying such as in freeze drying pellets.

When vials are standing on the shelf, the radiation from the surrounding surfaces that keep a higher temperature becomes more influential for the vials standing close to the perimeter of the shelf. These vials become warmer and dry faster than the samples in the center of the shelf. This effect is more pronounced at low pressures (< 10 Pa) than at higher pressures (10–100 Pa) due the relatively more important radiation at low pressures and at lower shelf temperatures. Important observation is that the vials standing in the middle of the shelf have a homogenous sublimation under all conditions. This suggest that to obtain a homogenous freeze drying, vials should be avoided to be placed close to the walls and door and avoid freeze drying at very low pressures and temperatures.

When vial holders are used, the samples remain exposed to the radiation but *via* the vial holder instead, and the conduction efficiency increases. Thus, the vial-to-vial variation is reduced. However, an inhomogeneous contact between the vial and vial-holder due to uneven vial surfaces and contamination may lead to quite variable sublimation even when using a vial-holder.

When oil was applied between surfaces, the contact and temperature control were further improved. However, the issue of the variable vial to vial-holder contact was also further amplified, and the final deviation in sublimation rate was further increased. The inhomogeneous results also resulted in a loss of reproducibility between experiments.

## Supplementary Information

Below is the link to the electronic supplementary material.

Supplementary file1 (DOCX 1641 kb)
