# Supplementary material for: Temperature and Heat Transfer Control During Freeze Drying. Effect of Vial Holders and Influence of Pressure
Source: Pharm Res. 2022 Aug 4;39(10):2597–606. doi: 10.1007/s11095-022-03353-4 (PMC9556401; doi:10.1007/s11095-022-03353-4)
Supplement: Supplementary file 1 — Supplementary file1 (DOCX 1641 kb) [file 11095_2022_3353_MOESM1_ESM.docx]

# Supplementary material

**S1 Sample temperature of individual oil sample for vials standing directly on shelf and inside an vial holder.**

**S2 Vial holder temperature with and without oil between shelf and vial holder at -10 ˚C and -40˚C.**

**S3 The average sample temperature for oil sample using the different setups.**

**S4 Sample temperature of water sample during sublimation.**


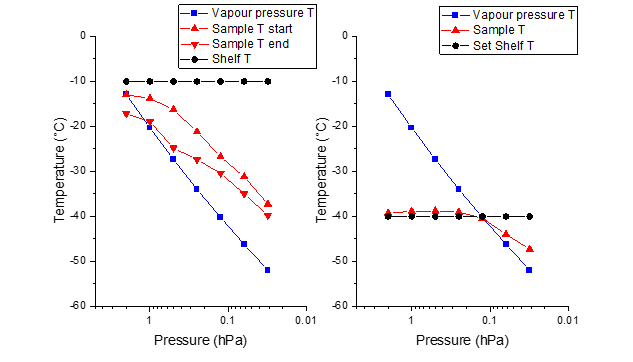


**S5 Measurements of sublimation rate of water as a function of pressure.**

Sublimation of water (gravimetric measurement),
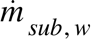
, heat flux,,
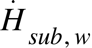
 and estimations of an apparent heat transfer coefficient, $K_{v,app}$, from eq. 1, between shelf and vial or between shelf over vial-holder to the vial. The temperature at the sublimation front, $T_{sf}$, is obtained from table data at the pressure of the dryer. $T_{shelf}$ refers to the temperature at the shelves. The sublimation entaply is determined at the temperature of the sublimation front using vapor pressure data and Clausius-Clapyreon’s equation. The apparent $K_{v}$ is estimated according to equation 1. All data from vials is an average of 54 samples.

|  |  |  |  | Vials at shelves | | | Vials in vial-holders | | | |
| --- | --- | --- | --- | --- | --- | --- | --- | --- | --- | --- |
| Pressure | *T_sf_* | *T_sf_-T_shelf_* | ${\Delta H}_{sub}$ | 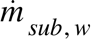*^a^* | 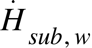 *^a^* | *K_v, app_^b^* | *J_Q,sub,w_^a^* | *J_H_,_sub,w_^a^* | *K_v, app_^b^* |  |
| (Pa) | (°C) | (°C) | kJ/kg | µg/s | mW | W/m^2^ K | µg/s | mW | W/m^2^ K |  |
| 3.12 | -52 | -37 | 2841 | 85.5 | 241 | 7.5 | 83.5 | 235 | 7.3 |  |
| 6.25 | -46 | -31 | 2840 | 91.6 | 258 | 10.0 | 96.0 | 271 | 10.5 |  |
| 12.5 | -40 | -25 | 2841 | 92.2 | 260 | 13.0 | 109.3 | 308 | 16.1 |  |
| 25 | -34 | -19 | 2841 | 93.4 | 264 | 19.2 | 118.9 | 336 | 27.1 |  |
| 50 | -27 | -12 | 2841 | 78.6 | 222 | 27.8 | 104.6 | 295 | 45.0 |  |

1. Per vial.
2. Per m^2^

**S6 The derivation of the expressions for the conductive heat transfer coefficient,** $\boldsymbol{K}_{\boldsymbol{v, cond}}$**, and the radiative heat transfer constant,** $\boldsymbol{c}_{\boldsymbol{rad}}$**. Equation [9] and [10].**

*Equations from the main text.*

The following equations are taken from the main text^[[1]](#footnote-1)^:

The heat sublimation heat flux balance of the sublimation experiment:

$\dot{H}_{sub,w}=\dot{H}_{cond,w}+\dot{H}_{rad,w}$ [2]

The heat flux balance of the oil experiment:

$\dot{H}_{cond,oil}=\dot{H}_{rad,oil}$ [3]

The equation defining the conductive heat flux as a function of the temperature difference over the bottom:

$\dot{H}_{cond,ref}=\Delta T_{b,ref}\cdot K_{v,cond}$ [4]

The conductive heat flux in the sublimation of water experiments:

$\dot{H}_{cond,w}=\Delta T_{b,w}\cdot K_{v,cond}$ [S4a]

The conductive heat flux in the oil experiments:

$\dot{H}_{cond,oil}=\Delta T_{b,oil}\cdot K_{v,cond}$ [S4b]

The total temperature difference between sublimation front and shelf distributed as a temperature drop over ice and over the bottom:

$T_{shelf}-T_{sf}=\Delta T_{ice}+\Delta T_{b,w}$ [5]

Temperature drop over the ice:

${\Delta T}_{\mathrm{ice}}=\frac{\dot{H}_{sub,w}}{(\lambda_{ice}\cdot\frac{A}{L})}$ [6]

Temperature elevation in the oil experiment:

$T_{\mathrm{oil}}-T_{\mathrm{shelf}}={\Delta T}_{b,oil}$ [7]

The equation defining the radiative heat flux as a function of the temperature difference between walls of the drying chamber and the sample:

$\dot{H}_{rad,ref}=\left\{ T_{wall}^{4}-T_{average, ref}^{4} \right\}\cdot c_{\mathrm{rad}}$ [8]

The radiative heat flux in the water experiments:

$\dot{H}_{rad,w}=\left\{ T_{wall}^{4}-T_{average, w}^{4} \right\}\cdot c_{\mathrm{rad}}$ [S8a]

The radiative heat flux in the oil experiments:

$\dot{H}_{rad,oil}=\left\{ T_{wall}^{4}-T_{average, oil}^{4} \right\}\cdot c_{rad}$ [S8b]

Where the average sample temperatures are:

$T_{average,w}=T_{sf}+\frac{\Delta T_{ice}}{2}$ [S8c]

$T_{average,oil}=T_{oil}$ [S8d]

*The derivation of the expression [9] and [10] in the main text.*

The derivation of the specific heat transfer coefficients for the conductive heat flow and for the radiative heat transfer is obtained by the following steps:

By combining the heat flux balance of sublimating water, equation [2], with the expressions of the conductive heat flux, equation [S4a], and of the radiative heat flux, equation [S8a]:

$\dot{H}_{sub,w}=K_{v, cond}\cdot\Delta T_{b,w}+c_{rad}\left\{ T_{wall}^{4}-T_{average,w}^{4} \right\}$ [S9]

By combining the heat flux balance of the elevated temperature in the oil experiment, equation [3], with the expressions of the conductive heat flux, equation [S4b], and of the radiative heat flux, equation [S8b], the following is obtained:

$K_{v, cond}\cdot\Delta T_{b,oil}=c_{rad}\left\{ T_{wall}^{4}-T_{average,oil}^{4} \right\}$ [S10]

From which *c_rad_* can be isolated:

$c_{rad}=\frac{K_{v, cond}\cdot\Delta T_{b,oil}}{T_{wall}^{4}-T_{average,oil}^{4}}$ [S11]

By using [S11] to replace $c_{rad}$ in expression [S9] we obtain an expression where $K_{v, cond}$can be obtained from $\dot{H}_{sub,w}:$

$\dot{H}_{sub,w}=K_{v, cond}\cdot\Delta T_{b,w}+K_{v, cond}\cdot\Delta T_{b,oil}\cdot\frac{\left\{ T_{wall}^{4}-T_{average,w}^{4} \right\}}{\left\{ T_{wall}^{4}-T_{average,oil}^{4} \right\}}$ [S12]

$K_{v, cond}=\frac{\dot{H}_{sub,w}}{\Delta T_{b,w}+\Delta T_{b,oil}\cdot\frac{\left\{ T_{wall}^{4}-T_{average,w}^{4} \right\}}{\left\{ T_{wall}^{4}-T_{average,oil}^{4} \right\}}}$ [S13]

Equation [S13] is equal to equation [9] in the main text.

$c_{rad}$ can be isolated from equation [S9] and become a function of $\dot{H}_{sub,w}$ and $K_{v, cond}$:

$c_{rad}=\frac{\dot{H}_{sub,w}-K_{v, cond}\cdot\Delta T_{b,w}}{T_{wall}^{4}-T_{average,w}^{4}}$ [S14]

[S14] is equal to expression [10] in the main text. Alternatively, $c_{rad}$ can be obtained from [S11].

*The definition of the symbols*

$\dot{H}_{sub,w}$ Experimentally observed heat flow when water (e. g. ice) is sublimated during freeze drying experiments [W], eq 2

$\dot{H}_{cond,w}$ Heat transfer through conduction, water experiments (from shelf to bottom of vial) [W] eq 2

$\dot{H}_{rad,w}$ Heat transfer through radiation, water experiments (from surounding warmer than the shelf) eq 2

$\dot{H}_{cond,oil}$ Heat transfer through conduction, oil experiments (from shelf to bottom of vial) [W] eq 3

$\dot{H}_{rad,oil}$ Heat transfer through radiation, water experiment (from surounding warmer than the shelf, e. g. walls and door) [W] eq 2

$T_{shelf}$ Temperature at shelf [K] eq 5. Set value in the experiments.

$T_{sf}$ Temperature at sublimation front [K] eq 5 (obtained as a function of pressure

using standard steam tables).

$T_{average,w}$ Average temperature of the water samples [K] eq S8 c

$T_{oil}$ Temperature of the oil experiments [K] eq S8 d, measured

$T_{average,oil}$ Average temperature of the oil samples [K] eq S8d

$T_{wall}$ Temperature of walls of the drying chamber [K] eq 8, estimated

$\Delta T_{b,w}$ Temperature difference between bottom of vial and shelf, water experiment [K] eq 5

$\Delta T_{b,oil}$ Temperature difference between bottom of vial and shelf, oil experiment [K] eq 7

$\Delta T_{ice}$ Temperature drop over the ice [K] eq 6

$\lambda_{ice}$ Heat conductivity of ice $\left[ \frac{W}{K\cdot m} \right]$eq 6

$K_{v, cond}$ Conduction constant for heat transfer from shelf to sample $\left[ \frac{W}{K} \right]$eq 4

$c_{rad}$ Radiation constant for heat transfer from walls to sample $\left[ \frac{W}{K^{4}} \right]$eq 8

$A$ Area of vial [m^2^] eq 6

$L$ Thickness of ice layer in vial [m] eq 6

**S7 Modelling of the heat transfer coefficient describing the heat flow from shelf to sample assuming conduction as described by the transition model.**

The estimation is done by using a slightly modified equation for the heat conduction between vials and shelf from the literature (Brülls and Rasmusson 2002 and Jousten 2016).

For vials on the shelf:

$K_{v,cond}=\frac{1}{\frac{L}{{A_{v} \Lambda}_{o}(\bar{T})}+\frac{1}{p A_{v}a_{A} \Lambda_{M0}\left( \frac{T_{0}}{\bar{T}} \right)^{0.5}}}$ [eq S1]

For vials in vialholders:

$K_{v,cond}=\frac{1}{\frac{L_{1}}{{A_{v1} \Lambda}_{o}(\bar{T})}+\frac{L_{2}}{{A_{v2} \Lambda}_{o}(\bar{T})}+\frac{1}{p A_{v1}a_{A} \Lambda_{M0}\left( \frac{T_{0}}{\bar{T}} \right)^{0.5}}+\frac{1}{p A_{v2}a_{A} \Lambda_{M0}\left( \frac{T_{0}}{\bar{T}} \right)^{0.5}}}$ [eq S2]

The constants and symbols:

|  |  |  |
| --- | --- | --- |
| *A_v_* | Area of the vial (d=11 mm) | 3.8·10^-4^ m^2^ |
| *A_v1_* | Area per vial of the vial holder on the shelf | 10.4·10^-4^ m^2^ |
| *A_v2_* | Area of vial in contact with the vial holder | 21.1·10^-4^ m^2^ |
| $a_{A}$ | Accommodation constant of water vapor at the aluminum oxide and the glass surface (assumed) | 1 |
| *L* | Distance between vial and shelf (estimated) | 250 µm |
| *L_1_* | Distance between vialholder and shelf (estimated) | 250 µm |
| *L_2_* | Distance between vialholder and vial (estimated) | 250 µm |
| $\bar{T}$ | Average temperature between vial bottom and shelf (here approximated) | $\bar{T}=\frac{T_{sf}+T_{shelf}}{2}$ [K] |
| $\Lambda_{o}\left( \bar{T} \right)$ | The conductivity of the water vapor as a function of temperature (extrapolated from the linear dependence between 40 - -20°C) | $\Lambda_{o}\left( \bar{T} \right)=7.07\cdot{10}^{-3}+\bar{T} 4\cdot{10}^{-5}$  [W/m K] |
| $\Lambda_{M0}$ | Molecular conductivity of the gas at 1 Pa and 0°C | 1.98 [W/Pa m^2^ K] |


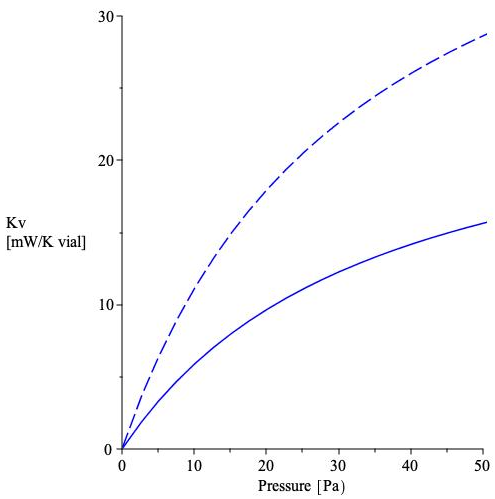


Heat transfer coefficient, *Kv,cond* estimated from equation S1 and S2. Continuous curve refers to vials on the shelf and dashed curve to vials in the vial holder.

**S8 Modelling of the sublimation heat flow originating the heat flow from shelf to sample using conduction as described by the transition model.**


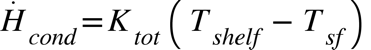


Where


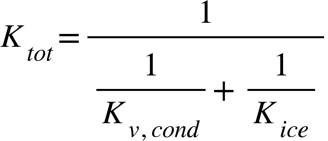
 and
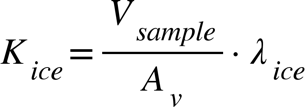


*l_ice_* is the conductivity of ice and *V_sample_* is the volume of the sample.


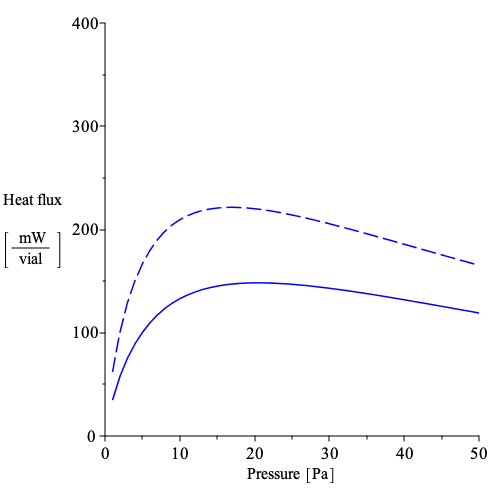


Modelled sublimation heat flow originating from the transfer of heat from the shelf to the vials assuming as described by the transition model given as a function of pressure. Solid line is vials on the shelf and dashed line is vials in the vial holder.

1. [#] refers to equations numbered in the main text. [S#] are numbers in the supplementary section. [S#a] refers to equations specifically referring to the sublimation of water (e.g. ice) experiments. [S#b] refers to equations specifically referring to the oil experiments. [S#c] refers to an un-numbered expression in the main text. [↑](#footnote-ref-1)
